# Multi-site feasibility and reproducibility study on UTE 3D phosphorous MRSI using novel rosette trajectory (PETALUTE)

**DOI:** 10.1002/mrm.30640

**Published:** 2025-07-22

**Authors:** Seyma Alcicek, Alexander R. Craven, Xin Shen, Mark Chiew, Ali Ozen, Stephen Sawiak, Ulrich Pilatus, Uzay E. Emir

**Affiliations:** 1 Goethe University Frankfurt, University Hospital, Institute of Neuroradiology, Frankfurt am Main, Germany; 2 University Cancer Center Frankfurt (UCT), Frankfurt am Main, Germany; 3 Frankfurt Cancer Institute (FCI), Frankfurt am Main, Germany; 4 Department of Clinical Engineering, Haukeland University Hospital, Bergen, Norway; 5 Department of Biological and Medical Psychology, University of Bergen, Bergen, Norway; 6 Radiology and biomedical imaging, University of California, San Francisco, California, USA; 7 Physical Sciences, Sunnybrook Research Institute, Toronto, Ontario, Canada; 8 Department of Medical Biophysics, University of Toronto, Toronto, Ontario, Canada; 9 Nuffield Department of Clinical Neurosciences, University of Oxford, Oxford, UK; 10 Department of Radiology, Medical Physics, Medical Center- University of Freiburg, Faculty of Medicine, University of Freiburg, Freiburg, Germany; 11 Department of Clinical Neurosciences, University of Cambridge, Cambridge, UK; 12 Department of Psychology, University of Cambridge, Cambridge, UK; 13 Weldon School of Biomedical Engineering, Purdue University, West Lafayette, Indiana, USA; 14 School of Health Sciences, Purdue University, West Lafayette, Indiana, USA; 15 Department of Radiology, University of North Carolina, Chapel Hill, North Carolina, USA; 16 The Lampe Joint Department of Biomedical Engineering, University of North Carolina, Chapel Hill, North Carolina, USA

**Keywords:** 3D ^31^P MRSI, compressed sensing, rosette K-space trajectory

## Abstract

**Purpose:**

This study aims (1) to implement a robust acquisition, fully automated reconstruction, and processing pipeline using a novel rosette k-space pattern for UTE ^31^P 3D MRSI and (2) to evaluate the clinical applicability and reproducibility at different experimental setups.

**Methods:**

A multi-center feasibility/reproducibility study was conducted for the novel UTE ^31^P 3D MRSI sequence with rosette petal trajectory (PETALUTE) at three institutions with different experimental setups (Siemens Prisma with volume head coil or surface coil, Siemens Biograph mMR with volume head coil). Five healthy subjects at each site were measured with an acquisition delay of 65 μs and a final resolution of 10 × 10 × 10 mm^3^ in 9 min. The measurement was repeated three times and averaged for the spectral analysis using the LCModel package. The potential for acceleration was assessed using compressed sensing on retrospectively undersampled data. Reproducibility at each site was evaluated using the inter-subject coefficient of variance.

**Results:**

This novel acquisition and advanced processing techniques yielded high-quality spectra and enabled the detection of the critical brain metabolites at three different sites with different hardware specifications. In vivo, feasibility with an acceleration factor of 4 in 6.75 min resulted in a mean Cramér-Rao lower bounds below 20% for phosphocreatine (PCr), adenosine triphosphate (ATP), phosphomonoesters (PME), and a mean coefficient of variation for ATP/PCr below 20%.

**Conclusion:**

We demonstrated that UTE ^31^P 3D rosette MRSI acquisition, combined with compressed sensing and LCModel analysis, allows clinically feasible, robust, high-resolution ^31^P MRSI to be acquired at clinical setups.

## 1 Introduction

Phosphorous (^31^P) MRS has shown much promise as a non-invasive molecular imaging tool since the earliest high-resolution NMR spectra were obtained from phosphate-containing metabolites in the early 70s.^1,2^ The first non-invasive molecular imaging followed these efforts via ^31^P MRS^3^ and its utilization in disease,^4^ wherein the technique provides a wide variety of molecular information. This includes adenosine triphosphate (ATP) and phosphocreatine (PCr), markers for deficits in energy metabolism, phosphomonoesters (PME, phosphorylethanolamine [PE] + phosphorylcholine [PC]) and phosphodiesters (PDE, glycerophosphoethanolamine [GPE]; glycerophosphocholine [GPC]), markers for cell synthesis and myelination, and nicotinamide adenine dinucleotide in oxidized (NAD^+^) and reduced (NADH) forms, reflecting cellular redox status^5,6^ and implicated in various signaling processes.^7^ Alterations in these metabolites may indicate impairment in energy storage and membrane synthesis or breakdown.^8–10^ Thus, ^31^P MRS can contribute to diagnosing and monitoring disease before structural/extracellular changes become apparent and can also allow measuring modulations in metabolites during physiological interventions.^11–15^

^31^P MRSI requires short TE due to the rather small T_2_ values for many metabolites of interest (e.g., T_2_(ATP)~40–75 ms^16^). This limits the application of single-voxel localization techniques, which require at least three consecutive frequency-selective RF pulses for localization. Only ISIS (Image Selected In Vivo Spectroscopy) can record localized data at short TE using a pulse-acquire method.^17^ However, it requires at least eight scans per cycle. Fortunately, the suppression of unwanted large signals from outside the region of interest is not as critical in ^31^P MRS as it is for ^1^H MRS, enabling the application of pulse-acquire MRSI sequences with a short delay between excitation and data acquisition. The common use of this technique with Cartesian phase-encoding in a clinically-feasible time suffers from a poor point spread function, hindering the accurate determination of metabolite concentrations in focal lesions.^18,19^ Also, acquisition delays (TE > 300 μs) with these methods result in phase and baseline distortion, causing operator errors in metabolite quantification.^5^

In summary, lower sensitivity, limited spectral–spatial resolution, and prolonged acquisition durations hamper the broader application of ^31^P MRSI in the clinical environment.^5^ As increasingly advanced MRSI acquisition technologies have become available,^20^ their improved resolution at comparable sensitivity can benefit ^31^P MRSI acquisition and quantification and facilitate robust clinical application of MRSI techniques. For instance, accelerated k-space trajectories^21–23^ and improved reconstruction approaches^22–24^ have mitigated long acquisition durations and improved SNR and resolution. Several offline data analysis tools have also demonstrated the feasibility of operator-independent metabolite quantification.^25,26^

This work intends to test the feasibility of a recently developed novel acquisition technique at 3T that addresses the challenges of speed, spatial resolution, first-order phase, and baseline distortion for ^31^P MRSI.^27^ The technique, which uses a novel rosette k-space pattern for UTE ^31^P 3D MRSI, was applied at three MR centers operating on 3T clinical Siemens MR scanners equipped with different hardware (surface or volume coil; standard-MR or PET-MR scanner). ^31^P MR metabolite spectra were obtained with a robust acquisition protocol and fully automated reconstruction and processing pipeline, which includes spectral analysis with LCModel. The performance of the proposed pipeline is assessed with LCModel data regarding spectra quality and signal intensities. Further acceleration with compressed sensing is demonstrated with a retrospectively undersampled rosette trajectory. Finally, the clinical applicability and reproducibility of UTE ^31^P 3D rosette MRSI readouts at different experimental setups are evaluated using inter-subject reproducibility.

## 2 Methods

This multi-center reproducibility study was conducted in accordance with the regulations of the local institutional human ethics committees. Five different healthy volunteers were scanned at each site. Volunteers underwent brain scans with a whole-body 3T MRI system. The contributing institutes, corresponding scanner configurations, and participant characterizations are summarized in Table 1.

### 2.1 UTE ^31^P 3D rosette MRSI acquisition

At all sites, a vendor-provided multi-plane isocenter localizer was measured for anatomical reference. The vendor-provided ^1^H B_0_ shimming procedure was performed with B_0_ shimming volume (adjustment volume) given in Table 1. The transmit voltage of the system for a pulse with a 90° flip angle was determined by recording the voltage at the maximum signal intensity in the manual adjustment protocol. The voltage for a desired excitation flip angle was calculated subsequent to calibration. The protocol was adjusted to use an Ernst flip angle of 30°, assuming that T_1_ of PCr at 3T is 2.6 s.^28^ While with the surface coil, which was used by protocol developers at Site 1, the required transmitter voltage for a 30° flip angle was achieved, Site 2 and Site 3 used volume coils and were limited to a maximum of 25° for the flip angle. Other acquisition parameters were the same between the sites and are given in Supporting Information.

**Figure 1:**
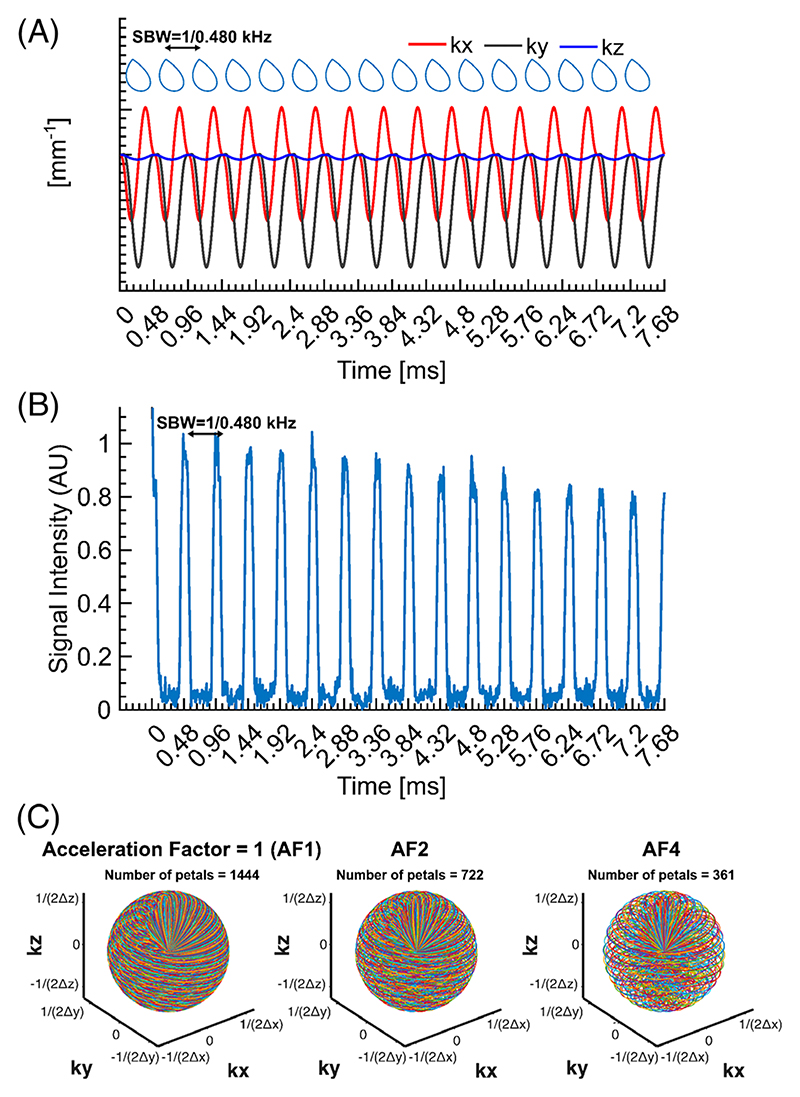
(A) The detailed k-space diagram illustrates the repetition of the same petal every 480 μs to achieve a spectral bandwidth of 2.083 kHz. (B) A representative k-space datum was acquired from a uniform phantom of inorganic phosphate. Only the first 16 repeats were illustrated for an average of 1444 petals. (C) An illustration of the 3D rosette k-space design with different acceleration factors (AF1, AF2, and AF4).

The 3D rosette MRSI k-space trajectory used equal radial and angular frequencies (ω_1_ = ω_2_ = 6500 rad/s) with each petal comprising 96 points, downsampled to 48 for reconstruction, resulting in an effective 100 kHz bandwidth; only the first 24 points were used to generate dual-echo images, and 1444 petals (80% of Nyquist-required 1810) were acquired in approximately 9 min. With 256 spectral points collected (effective spectral bandwidth = 2083 Hz, spectral resolution = 8.1 Hz, Figure 1A,B) and three signal averages, the total acquisition time for UTE ^31^P 3D rosette MRSI was 27 min (see Supporting Information for details).

### 2.2 Reconstruction and spectral analysis

Image reconstruction and post-processing steps of single-channel data were performed in MATLAB (Math-Works, USA). The nonuniform fast Fourier transform (NUFFT) was used to calculate the forward encoding transform of the acquired k-space data.^29^ A compressed sensing approach was used for image reconstruction, using total generalized variation as the sparsifying penalty.^30,31^ Specifically, the NUFFT modeled the Fourier transform of the acquired 3D *k*-space MRSI data on the trajectory for all time frames to generate 4D MRSI data with a final matrix size of 48 × 48 × 48 × 256. Retrospective undersampling of fully sampled datasets with compressed sensing was also analyzed for acceleration factors (AFs) of 2 and 4 to evaluate prospective acceleration (Figure 1C).^32^ We used the same total generalized variation regularization parameters across all AFs to avoid introducing bias across comparisons. For a robust spectral analysis, the resulting FIDs for all AFs were zero-filled to 1024 points and filtered with a Gaussian filter of 100 ms and 1 Hz Lorentzian line broadening. Automatic zero-order phase correction was performed by maximizing the sum of the real part of each spectrum.

The LCModel package was used to quantify the metabolite spectrum for each MRSI voxel^33^ (See Supporting Information for details on LCModel spectral fitting parameters). The SNR (SNR_LCModel_) and linewidth (LW_LCModel_) estimations of LCModel were used as spectral quality metrics.

### 2.3 Regional distributions of ^31^P metabolites and statistical analysis

The resulting MRSI resolutions were isotropic 10^3^ mm^3^ for Sites 1 and 3 and 12.5^3^ mm^3^ for Site 2. Mean metabolite levels in white matter and gray regions of interest (ROIs) were calculated.^34^ The workflow of post-processing steps is detailed in Supporting Information. For each site, the inter-subject variance was measured separately through the coefficient of variance (CoV). This analysis was performed on each voxel where PCr had Cramér-Rao lower bound (CRLB) (goodness of fit) values smaller than 20%. Metabolite ratios and spectral quality metrics for all voxels within an ROI were averaged together to calculate the ROI-specific values.

Mean and SD were determined from ROI-averaged metabolic ratios for every subject. Site-specific intersubject values were then averaged to calculate the inter-subject CoVs. Due to the different hardware setups, our acquisition protocol did not account for any statistical comparison between sites. As for reproducible research, we provide MATLAB scripts and k-space data to reproduce part of the results described in this article (https://purr.purdue.edu/projects/ismrm31pmrsi). To ensure robustness, data acquisitions were conducted with large 3D spatial coverage and vendor-provided B_0_ shimming procedure. Reconstruction and data processing were performed without any manual intervention (fully automated).

## 3 Results

**Figure 2:**
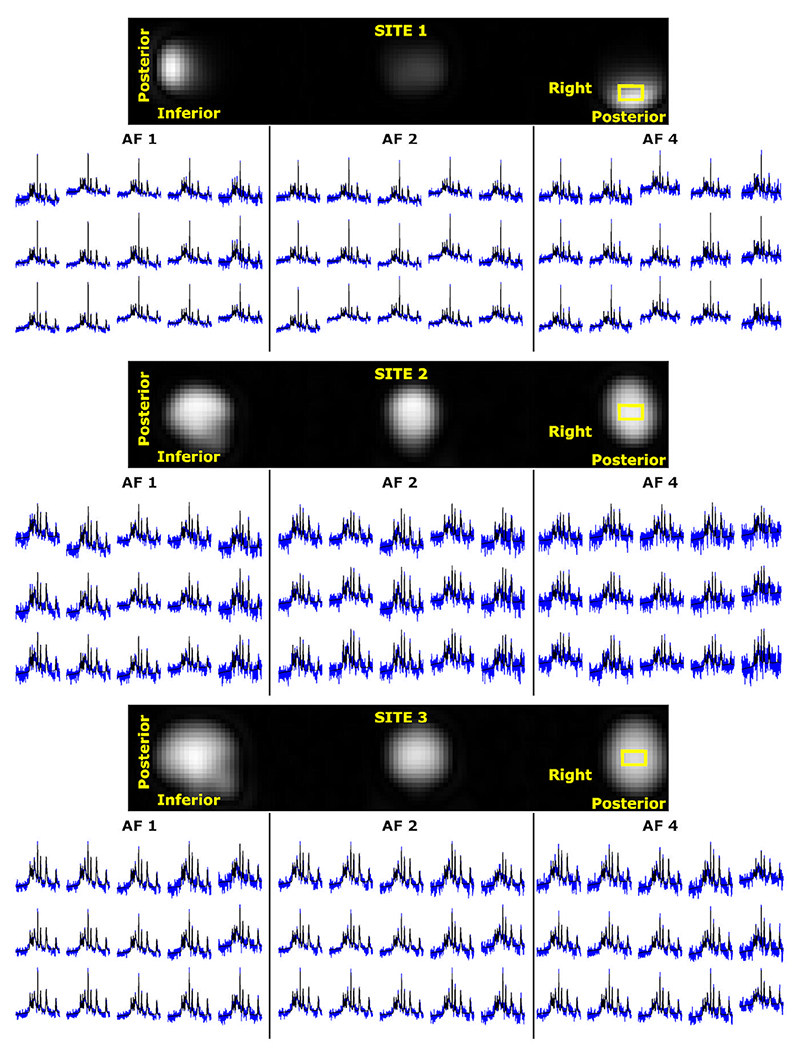
Extracted in vivo spectra (blue) from 15 voxels ofa5 × 3 grid with LCModel fit (black) for AF1, AF2, and AF4. The location of the 5 × 3 was illustrated as a yellow box on the first time-point of FID images.

Figure 2 shows the UTE ^31^P 3D rosette MRSI spectra on a 5 × 3 grid, with LCModel fits together with their first-time point of FID images. Due to hardware specifications, Site 1 resulted in coverage limited to the occipital cortex, while full brain coverage was achieved for Sites 2 and 3. The first-time point FID images generated images with structural information for sites 2 and 3. Even at a final resolution of 1 cm^3^ at 3T, spectra from three sites with different hardware setups are of sufficiently high quality for reliable LCModel analysis with full-k-space and retrospective AFs. The UTE ^31^P 3D rosette MRSI with an acquisition delay of 65 μs and processing pipeline with LCModel parameters results in a linear baseline and zero-order phase of the maximum of 25 degrees (Figures S5 and S6). As illustrated, similar baseline and phase distortions are also achieved with increased AFs, while the noise level rises due to undersampling.

**Figure 3:**
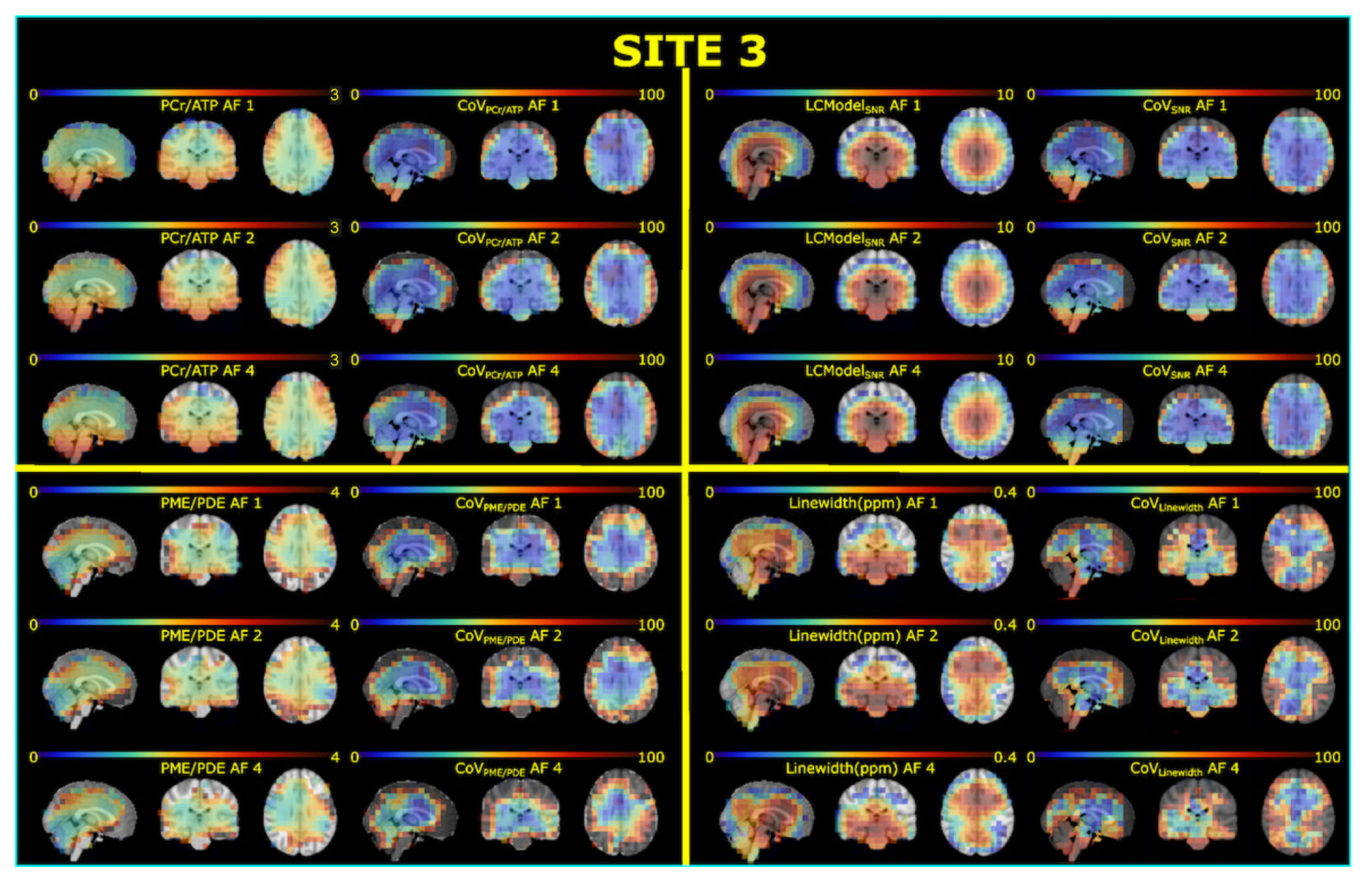
Metabolite ratios (PCr/ATP and PME/PDE), spectral quality metrics (SNR_LCModel_ and linewidth (LW)_LCModel_), and corresponding inter-subject CoVs maps for all AFs for Site 3. Maps are overlaid on the Montreal Neurological Institute-152 (MNI) template. Voxels resulting in a CoV higher than 100% were masked for each map.

**Figure 4:**
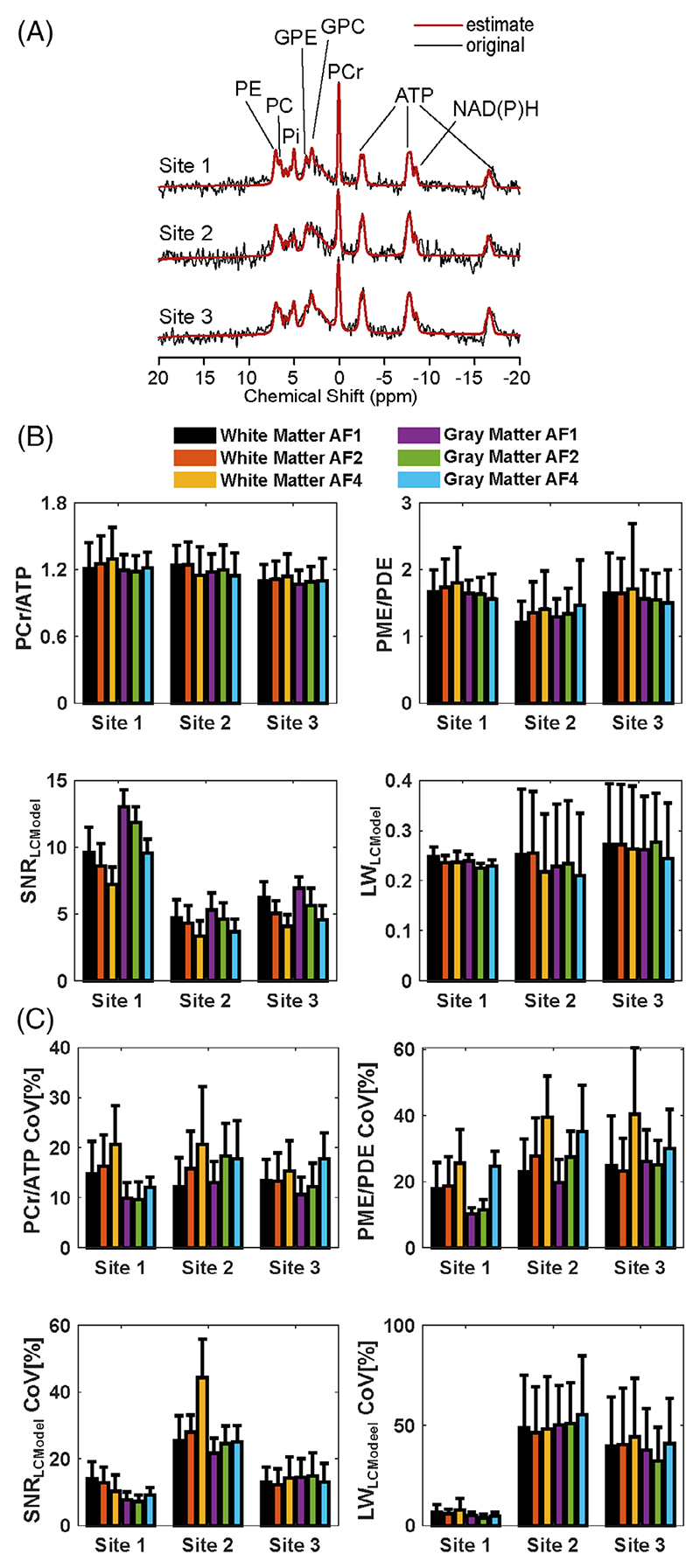
(A) Example ^31^P MR spectra from the middle of the ROI shown in Figure 2 for each site with the assignment of peaks to ^31^P-containing metabolites. The original signal is presented in black, and the LCModel spectral fit is in red. (B) The mean metabolite ratios (PCr/ATP and PME/PDE) and spectral quality metrics (SNR_LCModel_ and Linewidth (LW)_LCModel_) for each ROI across subjects for each site and AFs. (C) The mean inter-subject CoVs of metabolite ratios (PCr/ATP and PME/PDE) and spectral quality metrics (SNR_LCModel_ and Linewidth(LW)_LCModel_) for each ROI across subjects for each site and AFs.

The mean CRLB values of four reported metabolites (PCr, ATP, PME, and PDE) are lower than 25% in each ROI across subjects (Figure S7). Notably, the standard deviations of PDEs’ CRLBs are higher than the others, indicating PDE might not be reliably detected. Figures 3 and Figure S8 show high-spatial-resolution (10^3^ mm^3^) metabolite ratios, spectral quality metrics, and corresponding inter-subject CoV maps for all AFs for sites 3 and 1, respectively. The mean metabolite ratios for each ROI across subjects for each site and AFs are reported in Figure 4B. Overall, mean metabolite ratios of PCr/ATP and PME/PDE resulted in similar values across sites and ROIs. This does not change with increased undersampling (AFs). For instance, mean PCr/ATP in gray and white matter across subjects and sites for full-k-space acquisition are 1.14±0.06 and 1.17±0.06, respectively, whereas mean PME/PDE in gray and white matter are 1.49±0.18 and 1.50±0.25, respectively. Similar values are also observed for AF of 4, where mean PCr/ATP in gray and white matter were 1.14±0.06 and 1.17±0.09, respectively, whereas mean PME/PDE in gray and white matter were 1.49±0.18 and 1.50±0.25, respectively.

As for spectral quality metrics, there are significant performance differences between sites due to different setups. Data from Site 1 were taken with a surface coil. This provides an intrinsic higher SNR in the shown ROI. In addition, B_0_ shimming was different for the three sites. For the surface coil, it was focused on the area with the highest sensitivity of the coil, achieving good results for the shown ROI. For the other sites (2 and 3), it was optimized based on the adjustment volume, which was placed in the brain for Site 3 while covering a much larger area for Site 2. Thus, B_0_ homogeneity should be ranked best for Site 1 and worst for Site 2. The best indicator for a good shim is the linewidth of the PCr signal, where a smaller linewidth translates into a higher signal (assuming the same concentrations). Single representative spectra from the three sites with the assignments of peaks to ^31^P-containing metabolites are provided in Figure 4A, showing the highest PCr signal (relative to ATP) observed for Site 1, followed by Site 3, with Site 2 giving the lowest value. It should be noted that signal intensities (i.e., peak integrals) are not affected by the quality of the shim, since PCr/ATP values are comparable for the three sites. As expected, the SNR_LCModel_ decreased with increased undersampling for all sites. The LCModel parameter LW_LCModel_ gives almost the same linewidth for all sites, which would contradict the impression from Figures 4A and S6. However, LW_LCModel_, as a preliminary estimation obtained in the first processing step, might not provide the real linewidth. While data from the surface coils provide reasonable numbers for the standard deviation, the large SDs for Sites 2 and 3 (shown as whiskers in Figure 4B) indicate rather a poor performance in the estimation than the inter-subject variance. Data from the volume coils exhibit a higher MP hump, which might affect the preliminary linewidth estimate. The CRLB values for PCr shown in Figure S7 were determined in the final analysis step and should provide a better estimate of spectra quality. Lower CRLB values correlate well with increased SNR, as shown in Figure 3, and confirm the ranking determined above when discussing the representative spectra.

The mean inter-subject CoVs for metabolite ratios for each ROI and AF are reported in Figure 4C. As for metabolite ratios, overall, CoVs of PCr/ATP and PME/PDE resulted in similar values across sites and ROIs. CoVs for metabolite ratio increased with AF. For instance, the mean inter-subject CoV of PCr/ATP in gray and white matter of full-k-space acquisition for all sites are 11.22±1.67 and 13.45±1.22, respectively, whereas mean PME/PDE in gray and white matter are 18.75±8.02 and 22.02±3.58, respectively. Degraded CoVs are observed for AF of 4, where mean PCr/ATP in gray and white matter were 15.89±3.32 and 18.90±3.11, respectively, whereas mean PME/PDE in gray and white matter were 29.96±5.21 and 35.17±8.32, respectively. As for the mean SNR_LCModel_, the effect of different setups across sites echoed on CoVs. While CoVs remain lowest for Site 1 (surface coil), Sites 2 and 3 resulted in degraded CoVs for both SNR_LCModel_.

## 4 Discussion

This study demonstrated a robust acquisition protocol with a fully automated reconstruction and LCModel analysis pipeline for ^31^P MRSI. We implemented an identical UTE ^31^P 3D rosette MRSI sequence to assess reproducibility in healthy volunteers at three sites with different hardware specifications. This novel acquisition and advanced processing techniques yielded high-quality spectra and enabled the detection of the critical brain metabolites PCr, ATPs, PME, and PDE. In vivo feasibility with an AF of 4 in 6.75 min has been demonstrated with acceptable scan time and spatial resolution, indicating high spatial–temporal-spectral resolution in a relatively short time using compressed sensing. Reduced SNR at this AF can be compensated by slightly increasing the voxel size (i.e., FOV). In the presented study, a size of 12^3^ mm^3^ voxel would provide a similar gain in SNR as the averaging of three separate acquisitions. High spectral quality was consistently achieved with negligible baseline and phase distortion due to the unprecedented short acquisition delay for MRSI acquisition on a clinical scanner. As such, this work initiates standardization efforts for the long-awaited ^31^P MRSI on clinical scanners. Together, these results demonstrate the suitability of the proposed UTE ^31^P 3D rosette MRSI technique^35^ for clinical and experimental use.

### 4.1 Spectral quality and LCModel fitting

Several approaches to quantifying in vivo MR spectra, including ^31^P, have been described in Ref. 25,26. In particular, automated analysis of many ^31^P spectra of low SNR from a typical 3D-MRSI acquisition is challenging. Due to the choice of RF pulse and phase-encoding gradients in conventional acquisition strategies, ^31^P spectra inherited a first-order phase and baseline distortions. Mitigation of these artifacts requires operator-dependent evaluations. Because of the unprecedented acquisition delay of 65 μs, resulting in minimal phase and baseline distortions, this study could use the LCModel approach for automated analysis of ^31^P MR spectra.^25^ Overall, LCModel with UTE ^31^P 3D rosette MRSI resulted in automated evaluation, potentially minimizing methodological biases. Here, we showed that, regardless of the spectral quality degradation due to the retrospective undersampling, the automated LCModel analysis resulted in similar baseline and zero-order phases without operator manipulation. Thus, an automated voxel selection protocol was successfully implemented for all sites with different AFs.

### 4.2 Metabolite ratios

To mitigate potential biases originating from different hardware setups and short TR (350 ms), we chose to report metabolite ratios. In general, metabolite ratios reported in this study agree between sites and are comparable with those reported in previous studies. In particular, the PCr/ATP ratios measured in this study are in good agreement with those reporting PCr/ATP (α-ATP + β-ATP + γ-ATP)/3 and retrospective analysis of the ones that report discrete moieties (e.g., PCr/α-ATP or β-ATP).^5,22,25,36,37^ While similar mean PCr/ATP values are achieved with increased AFs, due to the noise amplification originating from undersampling, CoVs are increased. Similarly, PME/PDE values are in line between sites and with previous studies. In particular, this agreement holds firmly with studies that model the MP in their evaluations.^11,25,36^ As in PCr/ATP, similar degradation was observed on CoVs for PME/PDE with AFs, while metabolite ratios hold similar values across sites.

### 4.3 Clinical value

The different spectral quality, which is due to hardware and different adjustment volumes, has been discussed above. For clinical or research studies of the brain, volume coils should be preferred, since whole-brain coverage is frequently required. Comparison between sites 2 and 3 shows that the definition of an adjustment volume excluding areas with large inherent B_0_ inhomogeneities or focusing on the region with pathologies mitigates B_0_ inhomogeneities, improving the spectral quality regarding linewidth and peak intensity of the PCr signal. In addition, the lower performance of the gradient system in the PET/MRI scanner may contribute to slightly lower SNR on that platform.^38^ We found the covariance for PCr/ATP remains unaffected, indicating that the accuracy in determining the ATP signal intensity is the limiting factor. Given its broader linewidth, the ATP signal exhibits reduced sensitivity to shimming effects.

With well-determined PCr and ATP, the method could yield information on energy metabolism. Although the uncertainty of Pi signal intensity is rather high, its position, and thereby the intracellular pH, can be used for detecting pH changes. The same is valid for determining ion concentrations based on the position of the three ATP signals. As already discussed above, there is a drawback regarding accurate PDE quantification. While these metabolites provide valuable information for tumor diagnosis, their rather large T_1_ leads to lower signal intensity by partial saturation. Further, due to specific absorption rate (SAR) limits, the fast-sampling scheme does not allow for ^1^H decoupling, which would reduce PDE linewidth, increasing the accuracy of quantification. For tumor studies, the Ernst angle might be adjusted to the T_1_ of GPE (5.93 s)^39^ which should improve the detection of lipid-related metabolites but still gives a sufficient signal for PCr and ATP.

### 4.4 Limitations

While this study has demonstrated the feasibility and reproducibility of in vivo detection of ^31^P metabolites using novel accelerated acquisition and fully automated processing pipelines at three different hardware setups using 3T scanners from the same vendor, several factors affect the accuracy of the reported findings. Regarding sensitivity, the surface coil is superior to the volume coils for the occipital ROI, but incomplete brain coverage and inhomogeneous B_1_ limit the clinical applicability of this coil. In this study, vendor-provided standard 3D shimming performance resulted in higher CoV in metabolite estimations and spectral quality metrics for the sites due to the different shim volumes and receiver sensitivities of the coils. The receive sensitivity of the surface coil (Site 1) reduces the effective shim volume, resulting in improved reproducibility. For the volume coils, we observed a degradation in B_0_ homogeneity as the total shim volume increased. Adjustment volumes that encompass a targeted region of interest should be preferred. Alternatively, advanced whole-brain ^1^H shimming approaches can improve UTE ^31^P 3D rosette MRSI data.^40,41^

Here, to keep the protocol shorter, we measured MRSI with a short TR without B_1_ mapping, which is particularly necessary for the T_1_ correction of MRSI data acquired with the surface coil.^42^

## 5 Conclusions

In conclusion, we show that UTE ^31^P 3D rosette MRSI acquisition, combined with compressed sensing and LCModel analysis, allows fast, operator-independent, high-resolution ^31^P MRSI data to be acquired at 3T. Given the availability of 3T MRI scanners, the UTE ^31^P 3D rosette MRSI approach may have wide application in both clinical and research settings.

## Supplementary Material

Supplementary

## Figures and Tables

**Table 1:** Site-specific details for the multi-site UTE ^31^P 3D rosette-based MRSI study, including technical instrumentation, acquisition parameters, and participant characteristics.

Site no.	Institute	3T Siemens scannermodel	Maximum Gradient amplitude	Maximum slew rate	Coil	Healthy Volunteers (age, gender)	B_0_ Shimming volume (adjustment volume)	FOV	Special resolution	Used Maximum slew rate	Flip angle
1	Purdue University, USA	Magnetom Prisma	80 mT/m	200 mT/m/ms	Dual tuned surface coil, RAPID Biomedical	24±5 years (2 females)	240^3^ mm^3^	480^3^ mm^3^	10^3^ mm^3^	187.27 mT/m/ms	30°
2	Haukeland University Hospital, Bergen, Norway	Biograph mMR	40 mT/m	180 mT/m/ms	Dual tuned quadrature head coil, RAPID Biomedical	35 ±3 (1 female)	70 ×100 × 480 mm^3^	600^3^ mm^3^	12.5^3^ mm^3^	149.81 mT/m/ms	25°
3	University Hospital Frankfurt, Goethe University, Germany	Magnetom Prisma	80 mT/m	200 mT/m/ms	Dual tuned quadrature head coil, RAPID Biomedical	30 ±2 (2 females)	60^3^ mm^3^	480^3^ mm^3^	10^3^ mm^3^	187.27 mT/m/ms	25°

## Data Availability

We provide MATLAB scripts and k-space data to reproduce part of the results described in this article (https://purr.purdue.edu/projects/ismrm31pmrsi).
