# Supplementary material for: Multi-site feasibility and reproducibility study on UTE 3D phosphorous MRSI using novel rosette trajectory (PETALUTE)
Source: Magn Reson Med. Author manuscript; Available in PMC 2025 Dec 1. (PMC12501757; doi:10.1002/mrm.30640)
Supplement: Supplementary [file EMS211154-supplement-Supplementary.pdf]

Supporting Information: Multi-site Feasibility and Reproducibility Study on Ultrashort Echo Time 3D Phosphorous MRSI using novel Rosette Trajectory (PETALUTE)

UTE 31P 3D Rosette MRSI acquisition

The UTE ^{31}P 3D MRSI sequence with rosette k-space sampling[1] was measured with the following parameters: readout dwell time = 5 µs, TR = 350 ms, readout duration = 275 ms, and RF pulse duration = 50 µs. Field of view and achieved maximum slew rate in each site are given in **Table 1**. The ADC (bandwidth of 200 kHz) was turned on 10 µs after RF, and readout gradients were turned on 30 µs after ADC, resulting in an acquisition delay of 65 µs. These extra 6 ADC (6 x 5 µs) points were discarded before reconstruction (**Figure S1**). Before the MRSI data acquisition, 100 dummy TRs were run to reach the steady state.

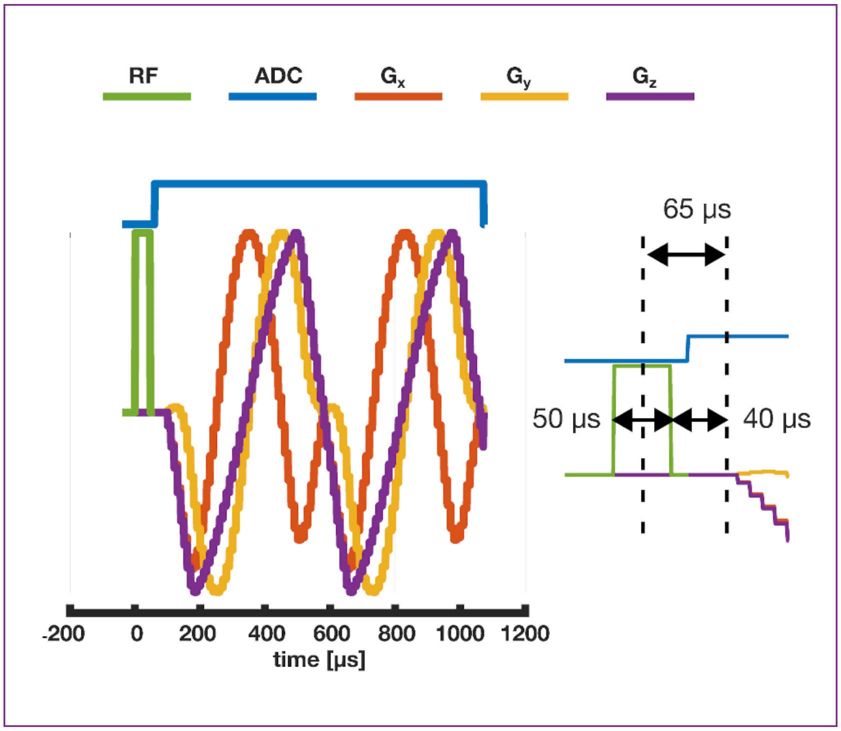

Figure S1 The detailed pulse diagram illustrates achieving an acquisition delay of 65 µs from the center of the RF pulse. ADC event starts ten us after the RF pulse. Readout gradients switch on after 6 ADC points (30 µs). The total delay for readout gradients from the end of the RF pulse is 40 µs.

The following equations were used for the 3D rosette MRSI k-space trajectory with a specific case where w_1 and w_2 are equal[1].

$$K_{xy}(t) = K_x(t) + iK_y(t)$$

$$K_{xy}(t) = K_{max} \times cos(\varphi) * sin(w_1 t) * e^{iw_2 t+\beta}$$

$$K_z(t) = \left(K_{max} * sin\ (\varphi)\right) * sin\ (w_1 t)$$

where $K_{max}$is the maximum extent of k-space, w_1 is the frequency of oscillation in the radial direction, w_2 is the frequency of rotation in the angular direction, φ determines the location in the z-axis, and β determines the initial phase in the angular direction.

According to this, each rosette petal was designed with 96 points per rosette with $w_1 = w_2$ of 6500 rad/s $\left(w_1 = \frac{\pi}{\left(dwelltime \times numberofpointsperpetal(N_{pp})\right)}\right)$. For the reconstruction, 96 points were downsampled to 48 by simply averaging the oversampled points, resulting in an effective bandwidth of 100 kHz. Since dual-echo images can be generated within a single acquisition with a manual separation at the middle of each data readout of the novel rosette acquisition, only the first half of the petal ($N_{pp}/2\ = 24$) was used for the reconstruction. According to Nyquist's criteria, the required number of petals (N_p) for a matrix size of 24 was calculated to be 1810 ($4 \times \pi \times 12^2$). Due to the efficient sampling of the rosette k-space pattern, only 80% of the required k-space, 1444, can be considered full-k-space acquisition[1]. The total acquisition time for UTE ^{31}P 3D Rosette MRSI was around 9 min (N_p x TR + dummy scans x TR= 505 s + 35 s). 256 spectral points (number of petal repeat or spectral points, N_{sp}= 256) were collected with an effective spectral bandwidth (SBW) of 2083 Hz for ^{31}P MRS, corresponding to a spectral resolution of 8.1 Hz (**Figure 1a, b**). Crusher gradients in all three directions were applied at the end of each readout gradient and before each excitation pulse.

The transmit voltage of the system for a pulse with a 90° flip angle was determined by recording the voltage at the maximum signal intensity in the manual adjustment protocol. The voltage for a desired excitation flip angle was calculated subsequent to calibration. The protocol was adjusted to use an Ernst flip angle of 30°, assuming that T_1 of PCr at 3T is 2.6s[2]. While with the surface coil, which was used by protocol developers at Site 1, the required transmitter voltage for a 30° flip angle was achieved, Site 2 and Site 3 used volume coils and were limited to a maximum of 25° for the flip angle.

Metabolite Level and Spectral Quality Metrics

The LCModel package was used to quantify the metabolite spectrum for each MRSI voxel[3]. The model spectra of PCr, α-ATP, β-ATP, γ-ATP, inorganic phosphate (P_i), PE, PC, GPE, GPC, pyridine nucleotides (NAD(P)H), membrane phospholipids (MP) and 2,3-diphosphoglycerate (DPG) were simulated using in-house MATLAB scripts with published ^{31}P chemical shifts and J-coupling constants[4]. Simulations with an ideal excitation pulse were performed using the same sequence timings (SBW and acquisition delay) as those on the 3 T systems in use **(Figure S1**). Since the quantifications were not corrected for T_1 and B_1 due to the scan time constraints, we chose to express results as metabolite ratios, PCr/(α-ATP+β-ATP+γ-ATP)/3 (PCr/ATP), and PME/PDE. In addition, to compare the performance of 3D MRSI at different sites, the SNR ($SNR_{LCModel}$) and linewidth ($LW_{LCModel}$) estimations of LCModel were used as spectral quality metrics. $SNR_{LCModel}$ is calculated using the peak height of the PCr singlet peak and the root-mean-square of its residual, and $LW_{LCModel}$ is calculated based on the preliminary analysis performed by LCmodel. Since the three metabolites (PCr, P_i, and NAD(P)H), defined by LCModel control parameter of CHUSE, were used in the preliminary analysis, $LW_{LCModel}$ should indicate the linewidth of PCr. The control file for the LCModel fitting is provided in the supporting information (**Figure S2**).

```
$LCMODL
TITLE='acc2_14_28_23'
OWNER='Purdue University'
HZPPPM=49.9815
NUNFIL=1024
DELTAT=0.00048008
NSIMUL=0
VITRO=.FALSE.
PPMCEN=0
PPMST=19.5
PPMEND=-19.5
FILBAS='P31.BASIS'
FILRAW='acc2_14_28_23.RAW'
FILPS='acc2_14_28_23.PS'
FILCOO='acc2_14_28_23.COORD'
LCOORD=9
FILPRI='acc2_14_28_23.PRINT'
LPRINT=0
NUSE1=3
CHUSE1(1)='PCr'
CHUSE1(2)='Pi'
CHUSE1(3)='NADP'
DOREFS=.FALSE.,.TRUE.
NREFPK(2)=1
PPMREF(1,2)=0
HZREF(1,2)=2*0.
ECCDON=.FALSE.
DEGZER=0
DEGPPM=0
SDDEGZ=5.
SDDEGP=0.15
DKNTMN=2*99.
RFWHM=3
XSTEP=5.
FWHMBA=0.049
NSIDMN=2
ALPBMN=108
ALPBMX=54000
ALPBPN=135
ALPBST=162
DESDSH=0.01
NAMREL='PCr'
CONREL=4.00
NEACH=0
NCOMBI=3
CHCOMB(1)='PE+PC'
CHCOMB(2)='GPE+GPC'
CHCOMB(3)='ATPa+ATPb+ATPg'
NOMIT=0
NRATIO=0
SHIFMN=-1
SHIFMX=1
PPMSHF=0
DEEXT2=7
DESDT2=7
NEXT2=1
CHEXT2(1)='MP'
ALEXT2(1)=400
CHEXT2(2)='UDP'
ALEXT2(2)=400
$END
```

Figure S2 The control file used in the LCModel analysis.

Regional Distributions of ^{31}P Metabolites and Statistical Analysis

The resulting MRSI resolutions were isotropic 10^3 mm^3 for Sites 1 and 3 and 12.5^3 mm^3 for Site 2. Each subject's MRSI slice and metabolite maps were determined in the subject's image space. The Montreal Neurological Institute-152 (MNI) template was downsampled to 10^3 mm^3 to match the MRSI resolution for coregistration purposes. Each subject's MRSI volumes of Sites 2 and 3 were coregistered to the MNI template using FMRIB's Linear Image Registration Tool (FLIRT) implemented

in the FMRIB's Software Library (FSL)[5]. Since the surface coil used in Site 1 resulted in limited brain coverage (occipital cortex), alignment of each subject's MRSI volume to MNI space was manually conducted using FSLeyes nudge[6] for that site. Afterward, mean metabolite levels in white matter and gray ROIs were calculated using fslstats[5]. Due to the different performances of the surface and volume coils used in this study, two sets of gray matter and white ROIs were identified for mean metabolite levels (**Figure S3**). The workflow of post-processing steps is summarized in **Figure S4**. The interactive AFNI view, showing slices and spectra graphs, was used to visualize the final results spectra[7].

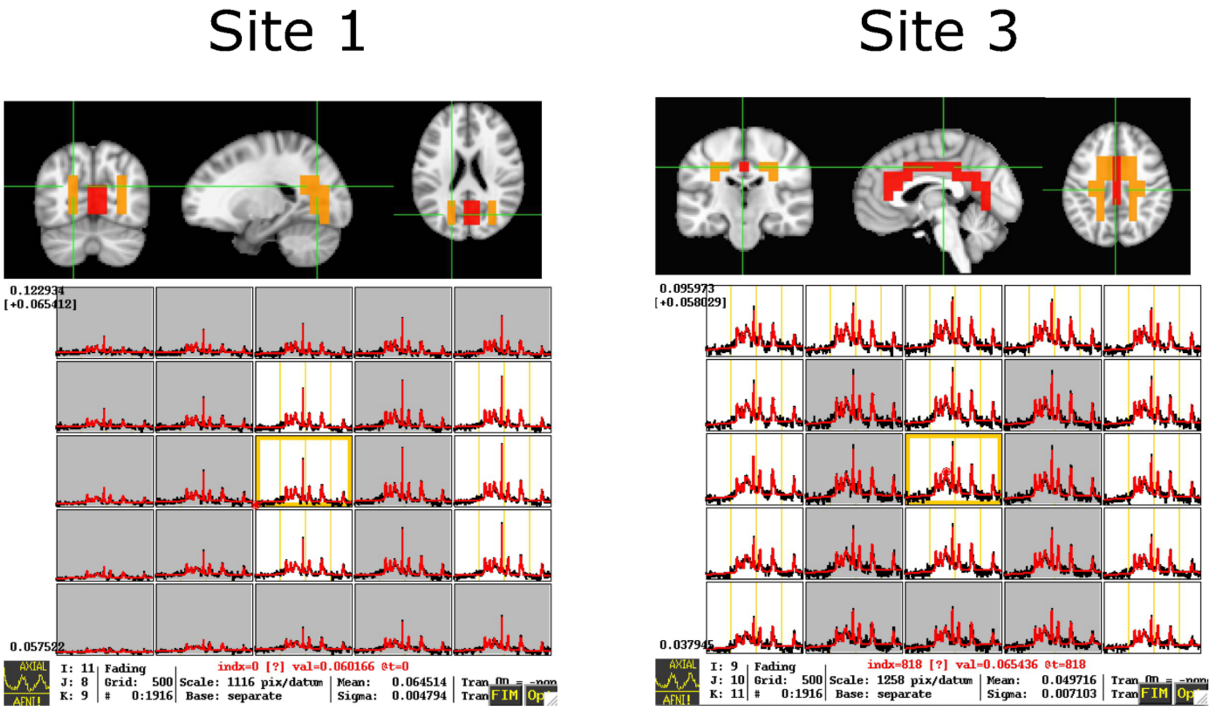

Figure S3 Gray (red) and white matter ROI masks for surface (Site 1) and volume coils (Site 2) overlaid on the Montreal Neurological Institute-152 template. The interactive AFNI view, showing slices and spectra graphs, was used to visualize the final result spectra.

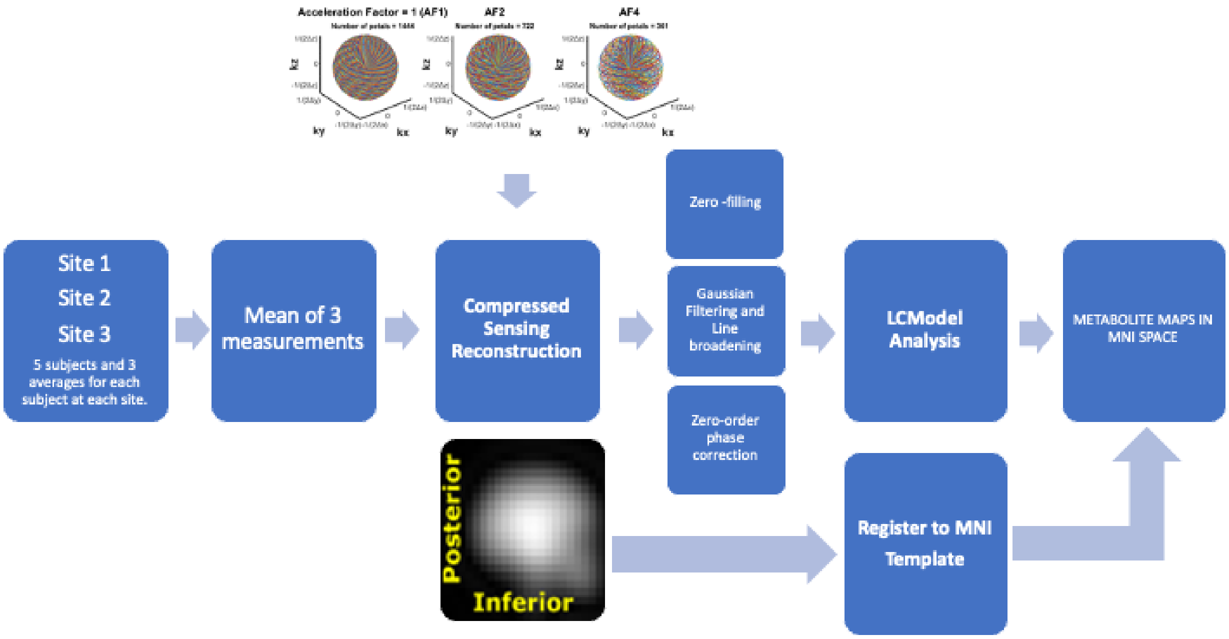

Figure S4 The workflow of post-processing steps with fully automated reconstruction, and spectra processing pipeline. Data were acquired from three different sites with five different subjects per site and three averages for each subject. After calculating the mean of three measurements, a compressed sensing reconstruction was performed using different acceleration factors (AF1, AF2, AF4) to undersample the k-space. Zero-filling, Gaussian filtering, line broadening, and zero-order phase correction were applied to the reconstructed spectral data. LCModel software was used for spectral analysis. The resulting metabolite maps were spatially normalized by registering to the MNI template. ROIs were chosen on the MNI template.

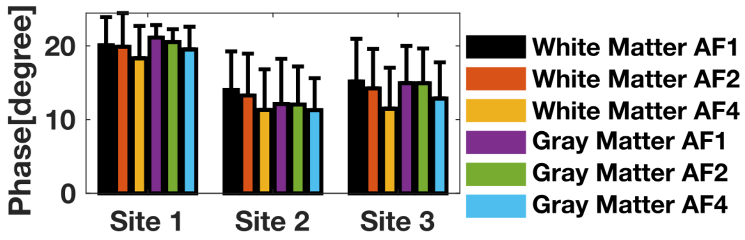

Figure S5 The mean zero-order phase estimations of the LCModel analysis for each ROI across subjects for each site and AF.

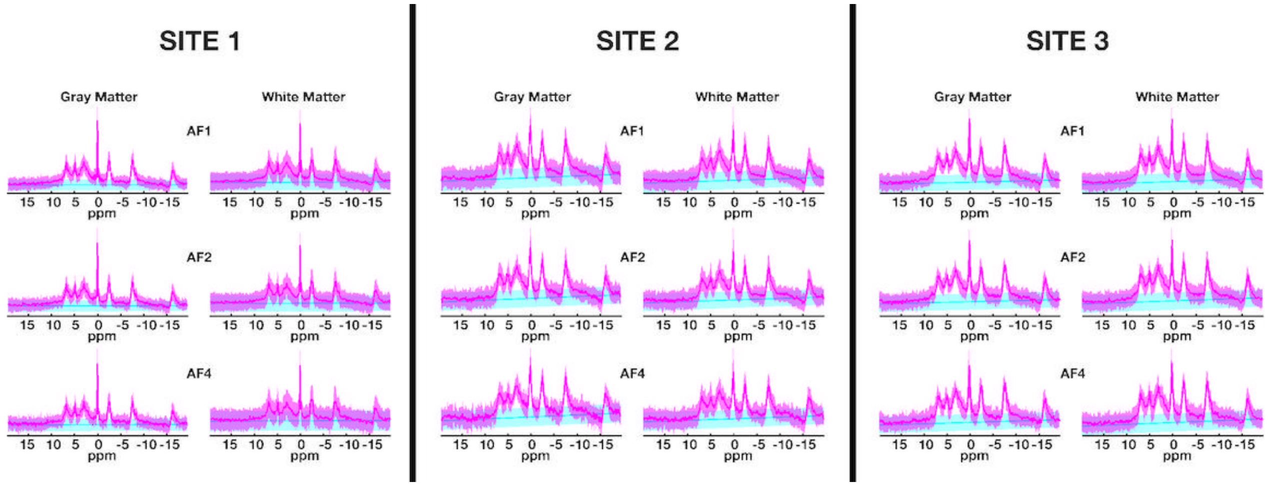

Figure S6 Mean (solid line) and ± standard deviation (shade) of ^{31}P MRS mean spectra (magenta) and LCModel baseline estimations (blue) in each ROI from all subjects for different AFs. Each column separated by black lines indicates different sites.

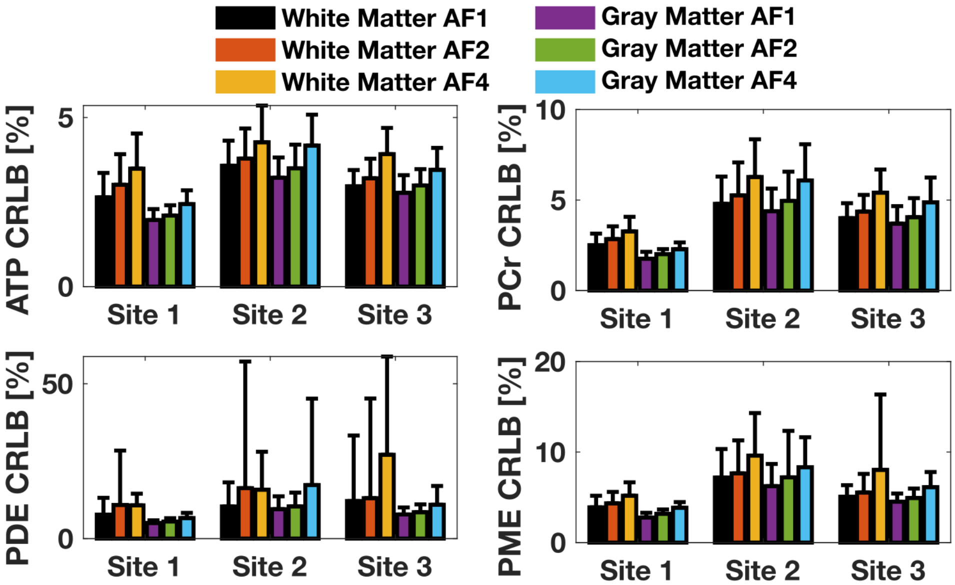

Figure S7 The mean CRLB for each ROI across subjects for each site and AF. The narrower chemical shift dispersion of ^{31}P MRS at lower magnetic fields (≤3T) results in an overlapping between MP and PDE signals. Including MP in the model may avoid an overestimated PDE and an underestimated PME/PDE, although substantial overlap of the signals leads to a higher CRLB standard deviation of PDE. Thus, the results of metabolite ratios must be cautiously interpreted.

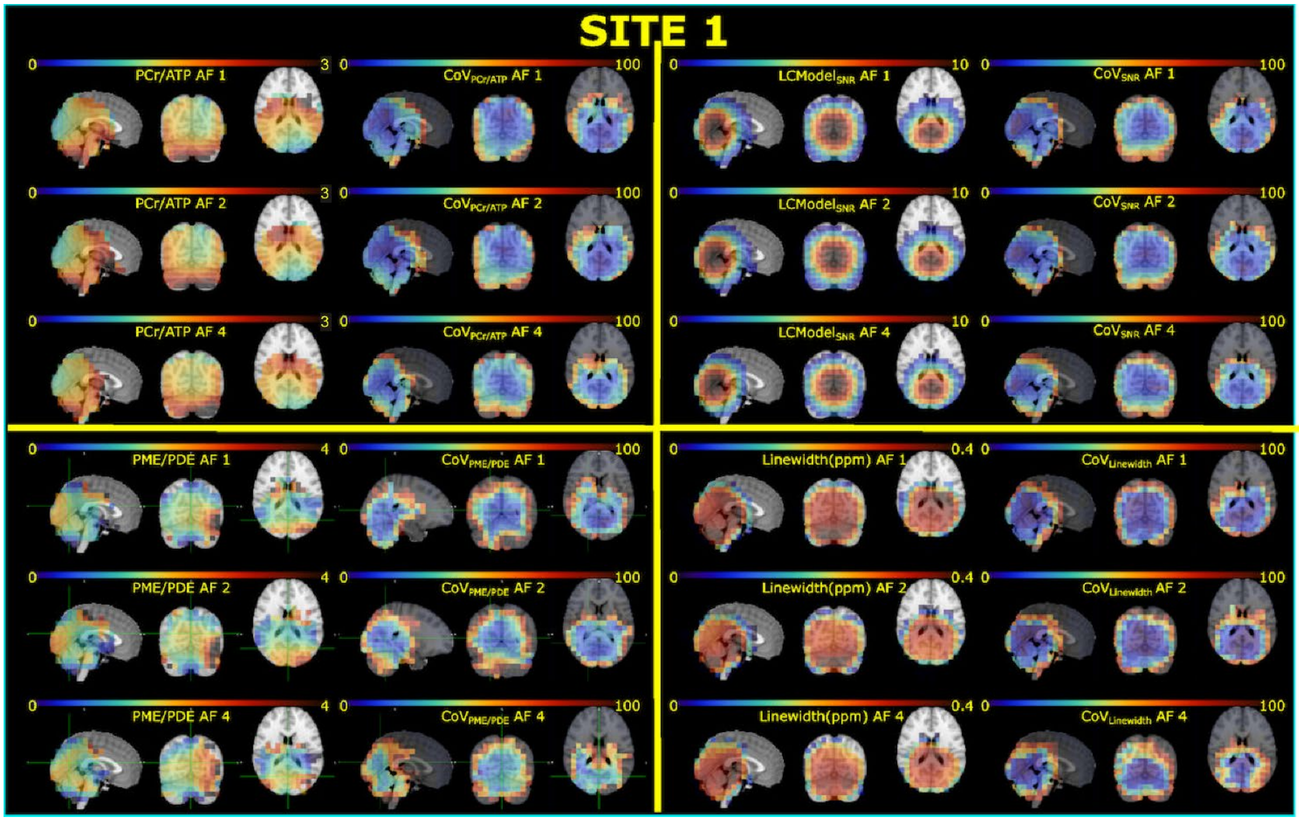

Figure S8 Metabolite ratios (PCr/ATP and PME/PDE), spectral quality metrics ($SNR_{LCModel}$ and Linewidth $(LW)_{LCModel}$), and corresponding inter-subject CoVs maps for all AFs for sites 1. Maps are overlaid on the Montreal Neurological Institute-152 (MNI) template. Voxels resulting in a CoV higher than 100 % were masked for each map.

References

1. Shen X, Özen AC, Sunjar A, et al. Ultra-short T2 components imaging of the whole brain using 3D dual-echo UTE MRI with rosette k-space pattern. *Magn Reson Med*. 2023;89(2):508-521. doi:10.1002/mrm.29451

2. Peeters TH, van Uden MJ, Rijpma A, Scheenen TWJ, Heerschap A. 3D 31P MR spectroscopic imaging of the human brain at 3 T with a 31P receive array: An assessment of 1H decoupling, T1 relaxation times, 1H-31P nuclear Overhauser effects and NAD+. *NMR Biomed*. 2021;34(5):e4169. doi:10.1002/nbm.4169

3. Provencher SW. Estimation of metabolite concentrations from localized in vivo proton NMR spectra. *Magnetic Resonance in Medicine*. 1993;30(6):672-679. doi:10.1002/mrm.1910300604

4. Deelchand DK, Nguyen TM, Zhu XH, Mochel F, Henry PG. Quantification of in vivo ^{31}P NMR brain spectra using LCModel. *NMR Biomed*. 2015;28(6):633-641. doi:10.1002/nbm.3291

5. Jenkinson M, Beckmann CF, Behrens TEJ, Woolrich MW, Smith SM. FSL. *Neuroimage*. 2012;62(2):782-790. doi:10.1016/j.neuroimage.2011.09.015

6. McCarthy P. FSLeyes. July 2020. doi:10.5281/zenodo.3937147

7. Cox RW. AFNI: software for analysis and visualization of functional magnetic resonance neuroimages. *Comput Biomed Res*. 1996;29(3):162-173. doi:10.1006/cbmr.1996.0014
